# The Reverse Shock Index Multiplied by Glasgow Coma Scale Score (rSIG) and Prediction of Mortality Outcome in Adult Trauma Patients: A Cross-Sectional Analysis Based on Registered Trauma Data

**DOI:** 10.3390/ijerph15112346

**Published:** 2018-10-24

**Authors:** Shao-Chun Wu, Cheng-Shyuan Rau, Spencer C. H. Kuo, Peng-Chen Chien, Hsiao-Yun Hsieh, Ching-Hua Hsieh

**Affiliations:** 1 Department of Anesthesiology, Kaohsiung Chang Gung Memorial Hospital, Chang Gung University and College of Medicine, Kaohsiung 83301, Taiwan; shaochunwu@gmail.com; 2 Department of Neurosurgery, Kaohsiung Chang Gung Memorial Hospital, Chang Gung University and College of Medicine, Kaohsiung 83301, Taiwan; ersh2127@cloud.cgmh.org.tw; 3 Department of Plastic Surgery, Kaohsiung Chang Gung Memorial Hospital, Chang Gung University and College of Medicine, Kaohsiung 83301, Taiwan; spenc19900603@gmail.com (S.C.H.K.); venu_chien@hotmail.com (P.-C.C.); sylvia19870714@hotmail.com (H.-Y.H.)

**Keywords:** Glasgow Coma Scale (GCS), Injury Severity Score (ISS), the rSI multiplied by GCS score (rSIG), Revised Trauma Score (RTS), shock index (SI), the Trauma and Injury Severity Score (TRISS), mortality

## Abstract

The reverse shock index (rSI) multiplied by Glasgow Coma Scale (GCS) score (rSIG), calculated by multiplying the GCS score with systolic blood pressure (SBP)/hear rate (HR), was proposed to be a reliable triage tool for identifying risk of in-hospital mortality in trauma patients. This study was designed to externally validate the accuracy of the rSIG in the prediction of mortality in our cohort of trauma patients, in comparison with those that were predicted by the Revised Trauma Score (RTS), shock index (SI), and Trauma and Injury Severity Score (TRISS). Adult trauma patients aged ≥20 years who were admitted to the hospital from 1 January 2009 to 31 December 2017, were included in this study. The rSIG, RTS, and SI were calculated according to the initial vital signs and GCS scores of patients upon arrival at the emergency department (ED). The end-point of primary outcome is in-hospital mortality. Discriminative power of each score to predict mortality was measured using area under the curve (AUC) by plotting the receiver operating characteristic (ROC) curve for 18,750 adult trauma patients, comprising 2438 patients with isolated head injury (only head Abbreviated Injury Scale (AIS) ≥ 2) and 16,312 without head injury (head AIS ≤ 1). The predictive accuracy of rSIG was significantly lower than that of RTS in all trauma patients (AUC 0.83 vs. AUC 0.85, *p* = 0.02) and in patients with isolated head injury (AUC 0.82 vs. AUC 0.85, *p* = 0.02). For patients without head injury, no difference was observed in the predictive accuracy between rSIG and RTS (AUC 0.83 vs. AUC 0.83, *p* = 0.97). Based on the cutoff value of 14.0, the rSIG can predict the probability of dying in trauma patients without head injury with a sensitivity of 61.5% and specificity of 94.5%. The predictive accuracy of both rSIG and RTS is significantly poorer than that of TRISS, in all trauma patients (AUC 0.93) or in patients with (AUC 0.89) and without head injury (AUC 0.92). In addition, SI had the significantly worse predictive accuracy than all of the other three models in all trauma patients (AUC 0.57), and the patients with (AUC 0.53) or without (AUC 0.63) head injury. This study revealed that rSIG had a significantly higher predictive accuracy of mortality than SI in all of the studied population but a lower predictive accuracy of mortality than RTS in all adult trauma patients and in adult patients with isolated head injury. In addition, in the adult patients without head injury, rSIG had a similar performance as RTS to the predictive risk of mortality of the patients.

## 1. Background

Identifying patients highly at risk of mortality is very important in managing the trauma patients. Among the many different prediction models for mortality outcomes of the trauma patients, the Trauma and Injury Severity Score (TRISS) remains the most commonly used algorithm [1,2]. The TRISS calculator determines the probability of survival from age, Injury Severity Score (ISS, an anatomical variable), Revised Trauma Score (RTS, a physiological variable), and the use of different coefficients for blunt and penetrating injuries. The Abbreviated Injury Scale (AIS) was used to grade the injury severity to an anatomical location on a six-point ordinal scale, ranging from minor (1 point), moderate (2 points), serious (3 points), severe (4 points), critical (5 points), to unsurvivable (6 points) [3], whereas the ISS is commonly used to grade the injury severity of trauma patients by the summation of squares of AIS score in the three most severe injuries of six predefined body regions [4]. The RTS is a weighted summation of coded variable values of the patient’s initial Glasgow Coma Scale (GCS) score and two vital signs [5], which include systolic blood pressure (SBP) and respiratory rate (RR) [5]. Although TRISS can predict the mortality outcome with high accuracy [6], TRISS can be only calculated using the information from all injured organs, which is not available on admission and is subjected to be changed after the admission; thus, its use in the prehospital stage or at the emergency department (ED) is limited [7].

The shock index (SI), a ratio of HR and SBP, had been developed to identify trauma patients in a hypovolemic shock [8]. A value of 0.7 represents normal SI, whereas SI of >1 is highly indicative of hemodynamic instability and mortality upon arrival at the ED [9,10]. An SI of ≥1 generally indicates an uncompensated shock state of the patient and resuscitation may be necessary [11,12]. An SI of ≥1 is also associated with higher mortality rate [13]. Therefore, we developed the reverse shock index (rSI), a ratio of SBP and HR, to indicate the hemodynamic condition of trauma patients [14,15,16,17]. The patient is in a potential shock when his (or her) SBP is decreased and lower than the HR (i.e., rSI of <1). This concept of rSI is intuitive utilizing two vital signs (SBP and HR) without any additional calculation and it can be used quickly in a prehospital scenario or a crowded ED [14,15,16,17]. We found that rSI of <1 was associated with poor outcome in the trauma patients and is helpful to identify the patients with a high risk to mortality, even when there is no notable hypotension [14,15,16,17].

In addition to hemorrhagic shock [18,19], traumatic brain injury [20,21] is another leading cause of mortality in trauma patients. The GCS score [22] is used to assess the level of consciousness at almost every ED worldwide and has been shown to be strongly associated with the probability of mortality in patients with traumatic brain injury [23,24]. Recently, a retrospective study from multicenters using registered data of 168,517 patients from the Japan Trauma Data Bank proposed that a new score, the rSI multiplied by GCS score (rSIG, i.e., rSIG = SBP/HR × GCS score), can be used to identify those trauma patients with a high risk for mortality and requirement of a blood transfusion within 24 h [25]. Therefore, we aimed to externally validate the accuracy of the rSIG in predicting the mortality outcomes in our cohort of trauma patients. Considering that rSIG uses physiological variables (SBP, HR, GCS) of trauma patients to predict mortality risk, SI, the ratio of two physiological variables (HR/SPB), RTS, the weighted sum of coded variable values of different physiological variables (GCS, SBP, and RR), and TRISS, the most commonly used prediction algorithm, would be used to compare the prediction outcome in this study.

## 2. Methods

### 2.1. Ethics Statement

Before reviewing the medical charts and the registered data in the Trauma Registry System of the hospital, this project had been approved (reference number: 201800875B0) by the institutional review board (IRB) of the Kaohsiung Chang Gung Memorial Hospital, the main referral level I trauma center in southern Taiwan [26,27]. Because of its character of a retrospective study design, the need for informed consent was waived off according to the regulation by IRB.

### 2.2. Study Population

This study included all adult patients aged ≥20 years who sustained a traumatic injury and were admitted in the hospital from 1 January 2009 to 31 December 2017. After excluding those who had burn injury (*n* = 726) and incomplete registered data (*n* = 47), 18,750 adult trauma patients were enrolled in the study and classified into two exclusive groups: patients with isolated head injury, presenting only head AIS of ≥2 (*n* = 2438), and those without head injury, with head AIS of ≤1 (*n* = 16,312). The selection of patients with head AIS ≥ 2 is based on the fact that head injury with head AIS = 1 are not fatal, and the mortality of patients with head AIS = 2, 3, 4, and 5 were 0.1%, 1.9%, 2.9%, and 31.1%, respectively [28]. The patients with multiple trauma in any other region of the body were excluded from the study; thus, the included patients were defined as having isolated head injury. The retrieved patient information included age; sex; SBP, HR, and GCS upon arrival at ED (if the patients were transferred after intubation or under sedation, prehospital GCS recorded by emergency medical service or the transferred hospital would be used); AIS over each body region; ISS; RTS; TRISS [29]; and, mortality in the hospital. The SI, rSI, and rSIG were calculated as the ratio of HR to SBP (SI = HR/SBP), ratio of SBP to HR (rSI = SBP/HR), and the score of rSI × GCS, respectively.

### 2.3. Statistical Analysis

Statistical analysis was performed using a SPSS software (version 22.0, IBM Corp., Armonk, NY, USA). In-hospital mortality of patients was the primary outcome of the study. We had used the Levene’s test to estimate the homogeneity of variance of continuous variables first. Then one-way analysis of variance with Games–Howell post-hoc test was used to evaluate the differences of continuous variables among patient groups. Mann-Whitney U-tests were used to analyze non-normally distributed data, which are presented as median with interquartile range (IQR, Q1–Q3). The values of continuous variables were expressed as mean ± standard deviation. The odds ratios (ORs) with 95% confidence intervals (CIs) of the categorical variables of gender and AIS were presented. By plotting specific receiver operating characteristic (ROC) curves, the SI, rSIG, RTS, and TRISS were evaluated to determine the best cutoff point that could predict the risk of mortality among these trauma patients. The accuracy of parameter in predicting the mortality outcomes was defined as an area under the curve (AUC) and was calculated based on the maximal Youden index (sensitivity + specificity − 1), to reflect the maximal correct classification accuracy. A nonparametric approach was performed to compare the accuracy of AUC ROC curves [30] using the roc & roc.test function in the pROC package in R3.3.3 (R Foundation for Statistical Computing, Vienna, Austria). Statistical significance was indicated when the *p*-value is <0.05.

## 3. Results

### 3.1. Patient Characteristics with All Types Trauma

Among the 18,750 patients who sustained with all types trauma, 18,248 survived and 502 died (Table 1). The patients who died were significantly older than those who survived and presented a significantly higher ISS, lower GCS, higher HR and RR, higher SI but lower rSI, and lower rSIG (median (Q1–Q3), 8.76 (5.56, 18.20) vs. 25.38 (19.22, 31.23); *p* < 0.001), RTS (5.03 (4.09, 6.90) vs. 7.84 (7.84, 7.84); *p* < 0.001), and TRISS (0.68 (0.45, 0.89) vs. 0.97 (0.94, 0.99); *p* < 0.001). Notably, the difference in SBP was not significant between the patients who died and those who survived. When compared with the surviving group, patients who died were significantly predominantly men. Regarding the AIS, patients who died had a significant higher score of AIS distribution in all body regions than those who survived.

### 3.2. Characteristics of Patients with Head Injury

Among the 2438 patients with head injury, 2209 survived and 229 died (Table 2). The patients who died were significantly older than those who survived and presented a significantly higher ISS, HR and SBP but lower GCS. No significant difference in SI and rSI was found between these two groups (both *p* = 0.111). The rSIG (10.69 (5.07, 20.43) vs. 25.67 (21.00, 31.09); *p* < 0.001), RTS (5.97 (4.09, 7.84) vs. 7.84 (7.84, 7.84); *p* < 0.001), and TRISS (0.72 (0.36, 0.93) vs. 0.98 (0.97, 1.00); *p* < 0.001) were significantly lower in patients who died than those who survived. Between the patients who survived and died, the difference in sex was not significant, and both of the variables were significantly different between the patients who survived and those who with all types of trauma. In terms of AIS, the patients who died had a significant higher score of AIS distribution in the head region than those who survived. 

### 3.3. Characteristics of Patients without Head Injury

As shown in Table 3, the patients who died were significantly older than those who survived and they presented a significantly higher ISS, lower GCS and SBP, higher HR and RR, higher SI, but lower rSI, and lower rSIG (10.69 (5.07, 20.43) vs. 25.67 (21.00, 31.09); *p* < 0.001), RTS (5.97 (4.09, 7.84) vs. 7.84 (7.84, 7.84); *p* < 0.001), and TRISS (0.72 (0.36, 0.93) vs. 0.98 (0.97, 1.00); *p* < 0.001). The patients who died were significantly predominantly men in comparison with the surviving group. In terms of AIS, the patients who died had a significant higher score of AIS distribution in all body regions than those who survived. 

### 3.4. Predictive Accuracy for Mortality

**Figure 1:**
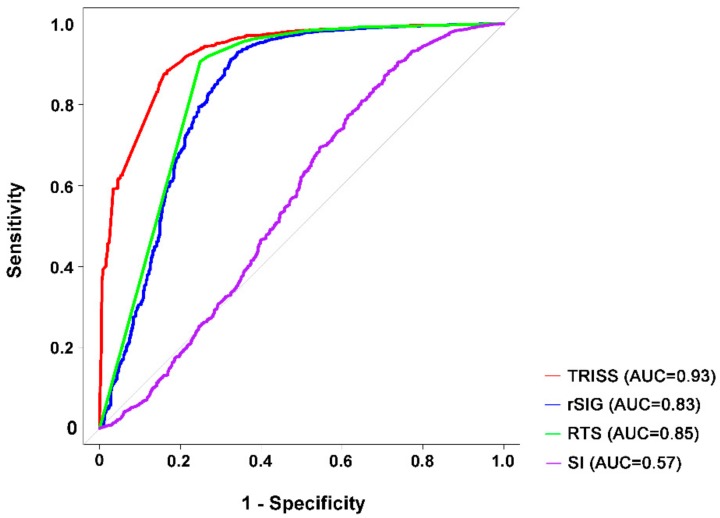
Area under the curve (AUC) of TRISS, rSIG, RTS, and SI in predicting the mortality of patients with all types of trauma.

Using the cutoff value of 14.8, the rSIG can estimate the probability of dying of all trauma patients with a sensitivity of 65.9% and specificity of 92.9% (Table 4). As shown in Figure 1, the predictive accuracy of SI (AUC 0.57) was the worst and significantly lower than all the other three predictive models. The predictive accuracy of rSIG (AUC 0.83) was significantly lower than that predicted by RTS (AUC 0.85, *p* = 0.02) and TRISS (AUC 0.93, *p* < 0.001). In addition, the predictive power of RTS was significantly lower than that of TRISS (*p* < 0.01). 

**Figure 2:**
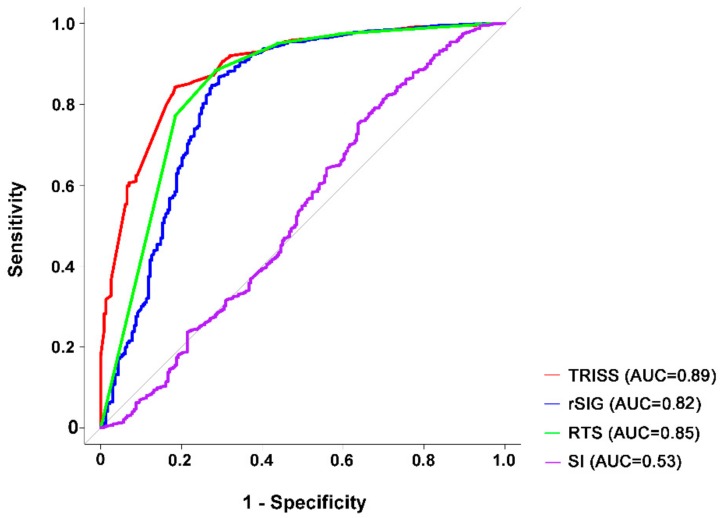
Area under the curve (AUC) of TRISS, rSIG, RTS, and SI in predicting the mortality of patients with isolated head injury.

In patients with head injury (Figure 2), the predictive accuracy of rSIG (AUC 0.82) was significantly lower than that predicted by RTS (AUC 0.85, *p* = 0.02) and TRISS (AUC 0.89, *p* < 0.001), and the predictive power of RTS was also significantly lower than that of TRISS (*p* < 0.01). Using the cutoff value of 14.8, the rSIG can estimate the probability of dying of trauma patients with head injury with a sensitivity of 70.7% and specificity of 86.8%. The predictive accuracy of SI (AUC 0.53) was significantly lower than all of the other three predictive models.

**Figure 3:**
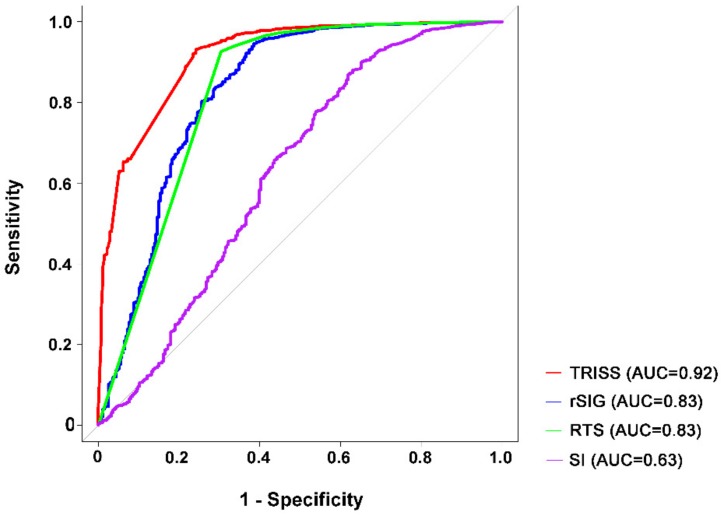
Area under the curve (AUC) of TRISS, rSIG, RTS, and SI in predicting the mortality of patients without head injury.

In patients without head injury (Figure 3), no difference in the predictive accuracy was observed between rSIG (AUC 0.83) and RTS (AUC 0.83, *p* = 0.97). Based on the cutoff value of 14.0, the rSIG can estimate the probability of dying of trauma patients without head injury with a sensitivity of 61.5% and specificity of 94.5%. Both rSIG and RTS had a predictive accuracy that was significantly lower than that of TRISS (AUC 0.92, both *p* < 0.001). The predictive accuracy of SI (AUC 0.63) was significantly lower than all other three predictive models.

We further explored the performance of these predictive models if they were applied on those patients with (Supplemental Table S1) or without (Supplemental Table S2) traumatic brain injury, which was defined as only head AIS ≥ 3 and found the results were similar to above presentation. In patients with isolated traumatic brain injury (Supplemental Figure S1), the predictive accuracy of rSIG (AUC 0.82) was significantly lower than that predicted by RTS (AUC 0.84, *p* = 0.02). In patients without traumatic brain injury (Supplemental Figure S2), no difference in the predictive accuracy was observed between rSIG (AUC 0.83) and RTS (AUC 0.82, *p* = 0.91). TRISS had the best and SI the worst predictive performance regardless in the patients with or without traumatic brain injury.

## 4. Discussion

In this study, we demonstrated that the predictive accuracy of rSIG was significantly lower than that by RTS in all trauma patients (AUC 0.83 vs. AUC 0.85, *p* = 0.02) and in patients with isolated head injury (AUC 0.82 vs. AUC 0.85, *p* = 0.02). However, in patients without head injury, no difference was found in the predictive accuracy between rSIG and RTS (AUC 0.83 vs. AUC 0.83, *p* = 0.97). 

Among these four models, TRISS is the best but SI is the worst model to predict the mortality of the trauma patients. Aside from the physiological variables (RTS), TRISS use additional information such as age, anatomical variable (ISS), and mechanism (blunt or penetrating) to predict the mortality outcome. Therefore, it is not surprising that TRISS had a better predictive accuracy than rSIG and RTS, which both only rely on the physiological changes in trauma patients. ISS and injury mechanism were strongly associated with the mortality outcome [6]. Age did matter in the prediction of mortality, when considering that older persons tend to have less sympathetic-responsive HR and higher SBP [31], which may lead to an increase in the false-negative values of SBP (even for SI or rSI) as age increases [32]. Old age had been reported to weaken the association of an SI of ≥1 and the 30-day mortality risk in all ED patients [33]. The predictive accuracy has also been reported to be highest for rSIG in predicting the survival in younger patients aged <55 years [25]. Among the patients aged ≥55 years, the value of rSIG divided by age (i.e., the indicator of rSIG/A) may indicate an in-hospital mortality better than that of rSIG [25].

The ISS value used to calculate TRISS cannot be obtained upon arrival at the ED or on admission; therefore, the use of rSIG or RTS is not intended to substitute TRISS in the prediction of mortality in trauma patients but rather to be used as a screening tool for high-risk patients at the ED. We had demonstrated that SI had a significantly worst predictive accuracy than all the other three models in this study. Unsurprisingly, the addition of more physiological variables in rSIG or RTS improve their predictive accuracy than SI. In this study, the predictive accuracy of rSIG was significantly lower than that by RTS in all trauma patients and in the patients with isolated head injury. Although both rSIG and RTS use GCS as a variable in predicting the mortality outcome, rSIG is calculated by multiplying the GCS score with SPB/HR, and RTS measures the sum of the coded values of GCS, SBP, and RR using the following formula: RTS = 0.9368 GCS + 0.7326 SBP + 0.2908 RR [5]. The RTS is heavily weighted toward the GCS to compensate major head injuries without multisystem injury or major physiological changes [5], which explain its higher predictive accuracy of mortality than rSIG in patients with isolated head injury. In contrast, in patients without head injury, the weight of GCS would be less important in predicting the mortality outcome, albeit the deterioration of consciousness may be found in some patients with profound shock [34]. However, the calculation of RTS is too complicated, preventing its easy use by the paramedics or at the ED. Moreover, RR, a component of RTS, is less reliable than other factors because it is heavily influenced by patient age, mechanism of injury, and the ventilation assistance or use of mechanical ventilation [35]. Notably, some patients in a shock status may have a disturbed consciousness, even if there was no associated head injury. Therefore, when considering that no difference was found in the predictive accuracy between rSIG and RTS in patients without head injury in this study, rSIG had similar performance in predicting mortality as RTS did in screening patients without head injury to identify subjects who are highly at risk of mortality at the ED. It is estimated that based on the cutoff value of 14.0 in this study, the rSIG can predict the probability of dying in trauma patients without head injury with a sensitivity of 61.5% and specificity of 94.5%.

This study had some limitations. First, because the study was a retrospective design study, some selection bias may be encountered. Second, the patients who were declared to be dead at the accident scene or upon arrival at the ED were not included in the registered database, and this might have resulted in selection bias in calculating the mortality rate. Third, the vital signs and GCS scores that were used in this study were those recorded upon patient’s arrival at the ED; however, such measurement is dynamic and it may be interfered by the resuscitation performed at prehospital scenario; thus, some bias in the calculation may happen. Fourth, the results of this study of rSIG in the patients with isolated head injury would not be generalized to all the patients with a head injury, while considering that some patients may have additional lethal injury into the other body region. Further, the study was limited to one trauma center, and the information obtained may limit its generalizability, and the cutoff values may also differ among countries or various trauma systems.

## 5. Conclusions

This study revealed that rSIG had a significantly higher predictive accuracy of mortality than SI in all studied population, but a lower predictive accuracy of mortality than RTS in all adult trauma patients and in adult patients with isolated head injury. In addition, in the adult patients without head injury, rSIG had similar performance as RTS to predictive the risk of mortality of the patients.

## Figures and Tables

**Table 1:** Characteristic variables of patients with all types of trauma.

Variables		Total	Survival		*p*-Value
		(*n* = 18,750)	No (*n* = 502)	Yes (*n* = 18,248)	
Age (years)		55 (38, 69)	65 (48, 77)	54 (37, 68)	<0.001
ISS		9 (4, 11)	25 (16, 29)	9 (4, 10)	<0.001
GCS		15 (15, 15)	6 (3, 14)	15 (15, 15)	<0.001
SBP (mmHg)		147 (127, 170)	153 (121, 184)	147 (127, 169)	0.122
HR (times/min)		85 (74, 97)	94 (77, 114)	84 (74, 96)	<0.001
RR (times/min)		18 (18, 20)	19 (18, 20)	18 (18, 20)	<0.001
SI		0.57 (0.47, 0.69)	0.62 (0.48, 0.84)	0.57 (0.47, 0.69)	<0.001
rSI		1.75 (1.44, 2.11)	1.60 (1.19, 2.10)	1.75 (1.45, 2.11)	<0.001
rSIG		25.46 (20.57, 30.98)	9.39 (5.25, 19.79)	25.64 (20.85, 31.09)	<0.001
RTS		7.84 (7.84, 7.84)	5.97 (4.09, 7.44)	7.84 (7.84, 7.84)	<0.001
TRISS		0.98 (0.97, 0.99)	0.70 (0.37, 0.92)	0.98 (0.97, 0.99)	<0.001
Sex, *n* (%)	Female	8150 (43.5%)	203 (35.3%)	7975 (43.7%)	<0.001
	Male	10,600 (56.5%)	325 (64.7%)	10,275 (56.3%)	
AIS (Head), *n* (%)	0	13,407 (71.5%)	87 (17.3%)	13,320 (73.0%)	<0.001
	1	1006 (5.4%)	12 (2.4%)	994 (5.5%)	
	2	388 (2.1%)	6 (1.2%)	382 (2.1%)	
	3	1280 (6.8%)	27 (5.4%)	1253 (6.9%)	
	4	2129 (11.4%)	122 (24.3%)	2007 (11.0%)	
	5	525 (2.8%)	235 (46.8%)	290 (1.6%)	
	6	15 (0.1%)	13 (2.6%)	2 (0.01%)	
AIS (Face), *n* (%)	0	16,003 (85.4%)	438 (87.3%)	15,565 (85.3%)	0.002
	1	873 (4.7%)	10 (2.0%)	863 (4.7%)	
	2	1829 (9.8%)	50 (10.0%)	1779 (9.8%)	
	3	45 (0.2%)	4 (0.8%)	41 (0.2%)	
AIS (Thorax), *n* (%)	0	16,376 (87.3%)	375 (74.7%)	16,001 (87.7%)	<0.001
	1	389 (2.1%)	11 (2.2%)	378 (2.1%)	
	2	592 (3.2%)	14 (2.89%)	578 (3.2%)	
	3	948 (5.1%)	51 (10.2%)	897 (4.9%)	
	4	419 (2.2%)	43 (8.6%)	376 (2.1%)	
	5	25 (0.1%)	7 (1.4%)	18 (0.1%)	
	6	1 (0.01%)	1 (0.2%)	0 (0.0%)	
AIS (Abdomen), *n* (%)	0	17,483 (93.2%)	436 (86.9%)	17,047 (93.4%)	<0.001
	1	86 (0.5%)	2 (0.4%)	84 (0.5%)	
	2	651 (3.5%)	27 (5.4%)	624 (3.4%)	
	3	366 (2.0%)	14 (2.8%)	352 (1.9%)	
	4	129 (0.7%)	19 (3.8%)	110 (0.6%)	
	5	35 (0.2%)	4 (0.8%)	31 (0.2%)	
AIS (Extremity), *n* (%)	0	5040 (26.9%)	316 (63.0%)	4724 (25.9%)	<0.001
	1	1163 (6.2%)	9 (1.8%)	1154 (6.3%)	
	2	7139 (38.1%)	89 (17.7%)	7050 (38.6%)	
	3	5358 (28.6%)	73 (14.5%)	5285 (29.0%)	
	4	43 (0.2%)	12 (2.4%)	31 (0.2%)	
	5	7 (0.04%)	3 (0.6%)	4 (0.02%)	
AIS (External), *n* (%)	0	17,027 (90.8%)	465 (92.6%)	16,562 (90.8%)	<0.001
	1	1613 (8.6%)	27 (5.4%)	1586 (8.7%)	
	2	85 (0.5%)	2 (0.4%)	83 (0.5%)	
	3	12 (0.06%)	0 (0.0%)	12 (0.1%)	
	4	3 (0.02%)	0 (0.0%)	3 (0.02%)	
	5	5 (0.03%)	4 (0.8%)	1 (0.01%)	
	6	5 (0.03%)	4 (0.8%)	1 (0.01%)	

AIS = abbreviated injury scale; GCS = Glasgow coma scale; ISS = injury severity score; rSI = reverse shock index; rSIG = rSI multiplied by GCS score; RTS = revised trauma score; SBP = systolic blood pressure; SI = shock index; TRISS = the trauma and injury severity score.

**Table 2:** Characteristics variables of patients with head injury.

Variables		Total	Survival		*p*-Value
		(*n* = 2438)	No (*n* = 229)	Yes (*n* = 2209)	
Age (years)		61 (45, 74)	68 (54, 78)	60 (44, 74)	<0.001
ISS		16 (9, 16)	25 (16, 25)	16 (9, 16)	<0.001
GCS		15 (11, 15)	4 (3, 9)	15 (13, 15)	<0.001
SBP (mmHg)		154 (134, 180)	158 (133, 197)	154 (135, 178)	0.01
HR (times/min)		84 (74, 97)	92 (75, 109)	84 (74, 96)	<0.001
RR (times/min)		18 (18, 20)	19 (17, 20)	18 (18, 20)	0.993
SI		0.55 (0.45, 0.66)	0.56 (0.45, 0.73)	0.55 (0.45, 0.66)	0.111
rSI		1.83 (1.51, 2.24)	1.77 (1.37, 2.23)	1.83 (1.52, 2.24)	0.111
rSIG		24.62 (17.75, 30.79)	8.76 (5.56, 18.20)	25.38 (19.22, 31.23)	<0.001
RTS		7.84 (6.90, 7.84)	5.03 (4.09, 6.90)	7.84 (7.84, 7.84)	<0.001
TRISS		0.97 (0.94, 0.99)	0.68 (0.45, 0.89)	0.97 (0.94, 0.99)	<0.001
Sex, *n* (%)	Female	906 (37.2%)	380 (35.4%)	16,866 (37.4%)	0.605
	Male	1532 (62.8%)	148 (64.6%)	1384 (62.7%)	
AIS (Head), *n* (%)	2	170 (7.0%)	2 (0.9%)	168 (7.6%)	<0.001
	3	693 (28.4%)	15 (6.6%)	678 (30.7%)	
	4	1253 (51.4%)	68 (29.7%)	1185 (53.6%)	
	5	310 (12.7%)	134 (58.5%)	176 (8.0%)	
	6	12 (0.5%)	10 (4.4%)	2 (0.1%)	

AIS = abbreviated injury scale; GCS = Glasgow coma scale; ISS = injury severity score; rSI = reverse shock index; rSIG = rSI multiplied by GCS score; RTS = revised trauma score; SBP = systolic blood pressure; SI = shock index; TRISS = the trauma and injury severity score.

**Table 3:** Characteristics variables of patients without head injury.

Variables		Total	Survival		*p*-Value
		(*n* = 16,312)	No (*n* = 273)	Yes (*n* = 16,039)	
Age (years)		54 (37, 68)	61 (42, 77)	54 (37, 67(	<0.001
ISS		9 (4, 9)	29 (18, 34)	8 (4, 9)	<0.001
GCS		15 (15, 15)	7 (3, 15)	15 (15, 15)	<0.001
SBP (mmHg)		146 (126, 168)	146 (107, 176)	146 (126, 168)	0.023
HR (times/min)		85 (75, 97)	96 (78, 117)	85 (75, 96)	<0.001
RR (times/min)		18 (18, 20)	20 (18, 20)	18 (18, 20)	<0.001
SI		0.58 (0.48, 0.70)	0.67 (0.51, 0.93)	0.58 (0.48, 0.69)	<0.001
rSI		1.73 (1.43, 2.09)	1.50 (1.07, 1.98)	1.73 (1.44, 2.09)	<0.001
rSIG		25.56 (20.83, 31.00)	10.69 (5.07, 20.43)	25.67 (21.00, 31.09)	<0.001
RTS		7.84 (7.84, 7.84)	5.97 (4.09, 7.84)	7.84 (7.84, 7.84)	<0.001
TRISS		0.98 (0.97, 1.00)	0.72 (0.36, 0.93)	0.98 (0.97, 1.00)	<0.001
Sex, *n* (%)	Female	7244 (44.4%)	351 (35.2%)	9359 (44.6%)	0.002
	Male	9068 (55.6%)	177 (64.8%)	8891 (55.4%)	
AIS (Head), *n* (%)	0	13,407 (82.2%)	87 (31.97%)	13,320 (83.1%)	<0.001
	1	1006 (6.2%)	12 (4.4%)	994 (6.2%)	
AIS (Face), *n* (%)	0	13,721 (84.1%)	214 (78.4%)	13,507 (84.2%)	<0.001
	1	717 (4.4%)	5 (1.8%)	712 (4.4%)	
	2	1829 (11.2%)	50 (18.3%)	1779 (11.1%)	
	3	45 (0.3%)	4 (1.5%)	41 (0.3%)	
AIS (Thorax), *n* (%)	0	13,992 (85.8%)	152 (55.7%)	13,840 (86.3%)	<0.001
	1	335 (2.1%)	5 (1.8%)	330 (2.1%)	
	2	592 (3.6%)	14 (5.1%)	578 (3.6%)	
	3	948 (5.8%)	51 (18.7%)	897 (5.6%)	
	4	419 (2.6%)	43 (15.8%)	376 (2.3%)	
	5	25 (0.2%)	7 (2.6%)	18 (0.1%)	
	6	1 (0.01%)	1 (0.4%)	0 (0.0%)	
AIS (Abdomen), *n* (%)	0	15,055 (92.3%)	208 (76.2%)	14,847 (92.6%)	<0.001
	1	76 (0.5%)	1 (0.4%)	75 (0.5%)	
	2	651 (4.0%)	27 (9.9%)	624 (3.9%)	
	3	366 (2.2%)	14 (5.1%)	352 (2.2%)	
	4	129 (0.8%)	19 (7.0%)	110 (0.7%)	
	5	35 (0.2%)	4 (1.5%)	31 (0.2%)	
AIS (Extremity), *n* (%)	0	2743 (16.8%)	94 (34.4%)	2649 (16.5%)	<0.001
	1	1022 (6.3%)	2 (0.7%)	1020 (6.4%)	
	2	7139 (43.8%)	89 (32.6%)	7050 (44.0%)	
	3	5358 (32.9%)	73 (26.7%)	5285 (33.0%)	
	4	43 (0.3%)	12 (4.4%)	31 (0.2%)	
	5	7 (0.04%)	3 (1.1%)	4 (0.02%)	
AIS (External), *n* (%)	0	14,818 (90.8%)	246 (90.1%)	14,572 (90.95%)	<0.001
	1	1384 (8.5%)	17 (6.2%)	1367 (8.5%)	
	2	85 (0.5%)	2 (0.7%)	83 (0.5%)	
	3	12 (0.1%)	0 (0.0%)	12 (0.1%)	
	4	3 (0.02%)	0 (0.0%)	3 (0.02%)	
	5	5 (0.03%)	4 (1.5%)	1 (0.01%)	
	6	5 (0.03%)	4 (1.5%)	1 (0.01%)	

AIS = abbreviated injury scale; GCS = Glasgow coma scale; ISS = injury severity score; rSI = reverse shock index; rSIG = rSI multiplied by GCS score; RTS = revised trauma score; SBP = systolic blood pressure; SI = shock index; TRISS = the trauma and injury severity score.

**Table 4:** The best cutoff point with its sensitivity and specificity that could predict the risk of mortality among the trauma patients.

Variables		Best Cutoff Point	Sensitivity (%)	Specificity (%)	AUC
TRISS	All patients	1	88.4	83.3	0.93
	Head injury	0.9	84.2	81.7	0.89
	No head injury	0.9	93.1	75.8	0.92
rSIG	All patients	14.8	92.9	65.9	0.83
	Head injury	14.8	86.8	70.7	0.82
	No head injury	14	94.5	61.5	0.83
RTS	All patients	7.7	90.7	75.1	0.85
	Head	6.5	88.5	71.6	0.85
	No Head	7.7	92.7	69.6	0.83
SI	All patients	0.8	88.3	28.5	0.57
	Head injury	0.7	75.4	36.2	0.53
	No head injury	0.8	86.9	38.1	0.63

rSIG = rSI multiplied by GCS score; RTS = revised trauma score; SI = shock index; TRISS = the trauma and injury severity score.

## Supplementary Materials

The following are available online at http://www.mdpi.com/1660-4601/15/11/2346/s1, Figure S1: Area under the curve (AUC) of TRISS, rSIG, RTS, and SI in predicting the mortality of patients with isolated traumatic brain injury. Figure S2: Area under the curve (AUC) of TRISS, rSIG, RTS, and SI in predicting the mortality of patients without traumatic brain injury. Table S1: Characteristics variables of patients with isolated traumatic brain injury (only head AIS ≥ 3). Table S2: Characteristics variables of patients without traumatic brain injury.
