# Supplementary material for: The Reverse Shock Index Multiplied by Glasgow Coma Scale Score (rSIG) and Prediction of Mortality Outcome in Adult Trauma Patients: A Cross-Sectional Analysis Based on Registered Trauma Data
Source: Int J Environ Res Public Health. 2018 Oct 24;15(11):2346. doi: 10.3390/ijerph15112346 (PMC6266192; doi:10.3390/ijerph15112346)
Supplement: Supplementary file 1 [file ijerph-15-02346-s001.pdf]

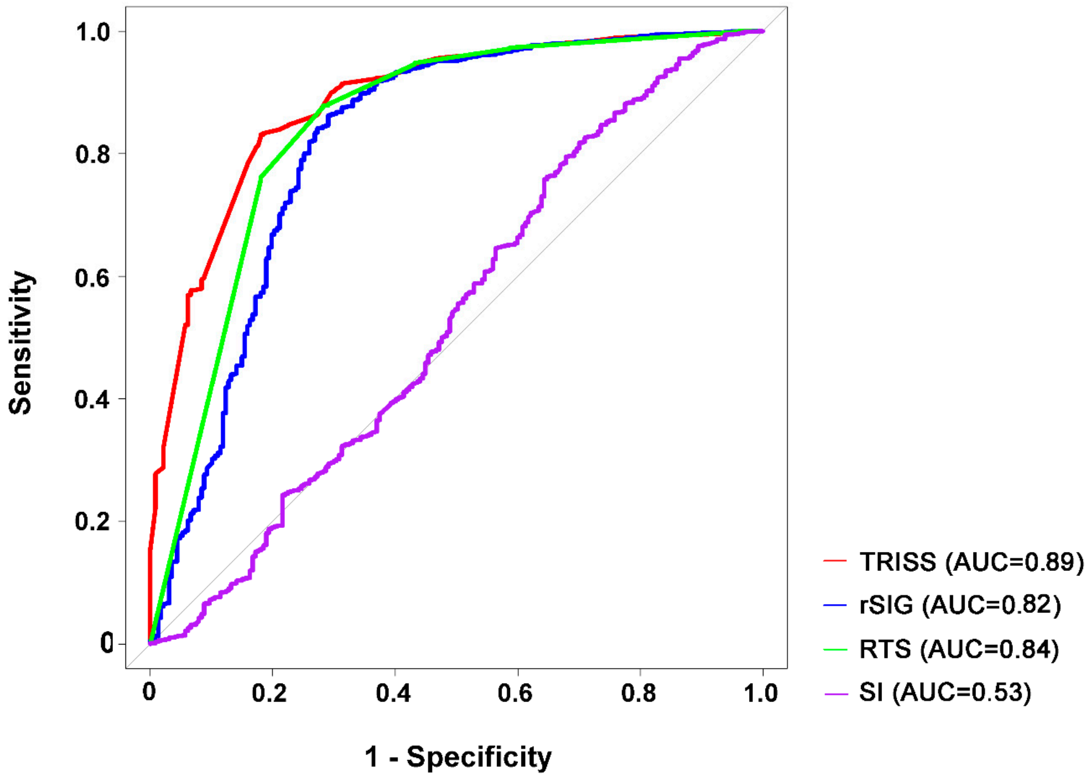

Figure S1. Area under the curve (AUC) of TRISS, rSIG, RTS, and SI in predicting the mortality of patients with isolated traumatic brain injury.

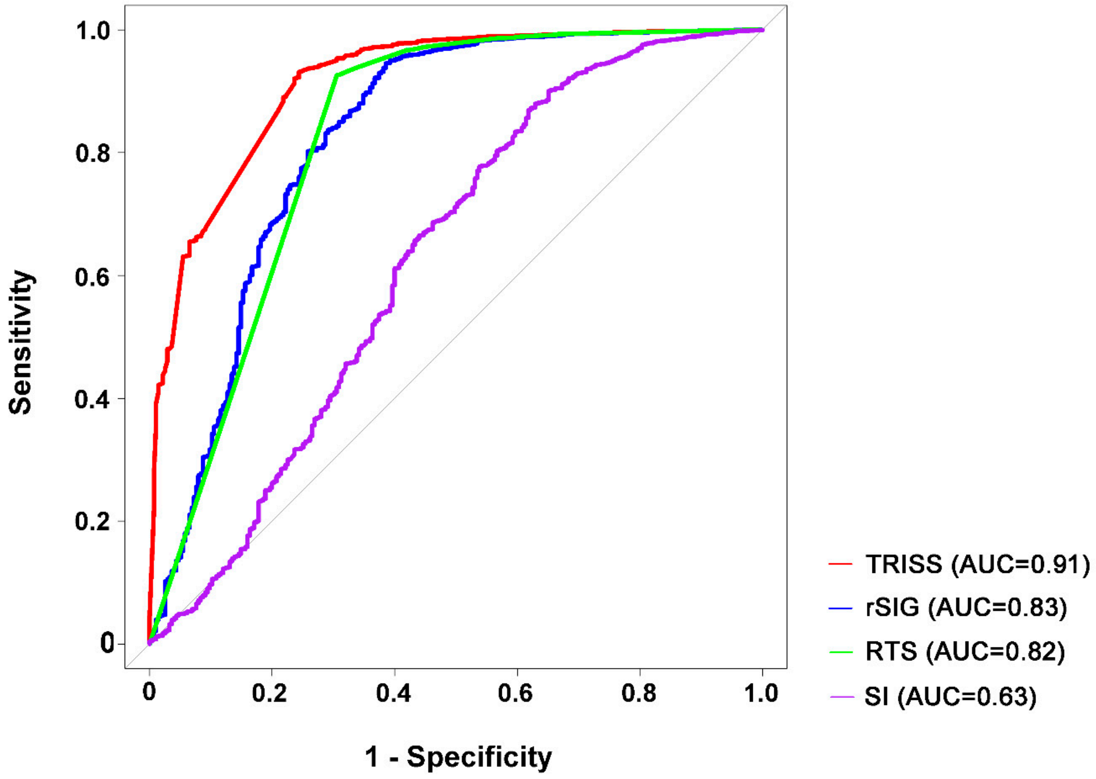

Figure S2. Area under the curve (AUC) of TRISS, rSIG, RTS, and SI in predicting the mortality of patients without traumatic brain injury.

Table S1. Characteristics variables of patients with isolated traumatic brain injury (only head AIS ≥ 3).

**Variables**		**Total (n = 2,268)**	**Survival**		***P*-value**
			**No (n = 227)**	**Yes (n = 2,041)**	
Age (years)		61 [45, 74]	68 [54, 78]	61 [45, 74]	<0.001
ISS		16 [9, 16]	25 [16, 25]	16 [10, 16]	<0.001
GCS		15 [11, 15]	4 [3, 9]	15 [13, 15]	<0.001
SBP (mmHg)		154 [134, 180]	158 [134, 198]	154 [135, 180]	0.016
HR (times/min)		84 [74, 97]	92 [75, 109]	84 [74, 96]	<0.001
RR (times/min)		18 [18, 20]	19 [17, 20]	18 [18, 20]	0.831
SI		0.55 [0.45, 0.66]	0.56 [0.45, 0.73]	0.54 [0.44, 0.65]	0.103
rSI		1.83 [1.51, 2.24]	1.78 [1.37, 2.23]	1.85 [1.53, 2.26]	0.103
rSIG		24.62 [17.75, 30.79]	8.76 [5.54, 17.96]	25.36 [19.19, 31.23]	<0.001
RTS		7.84 [6.90, 7.84]	5.03 [4.09, 6.90]	7.84 [7.84, 7.84]	<0.001
TRISS		0.97 [0.94, 0.99]	0.68 [0.45, 0.89]	0.97 [0.94, 0.99]	<0.001
Sex	Female	848 (37.4%)	80 (35.2%)	768 (37.6%)	0.527
	Male	1,420 (62.6%)	147 (64.8%)	1,273 (62.47%)	
AIS (Head)	3	693 (30.6%)	15 (6.6%)	678 (33.2%)	<0.001
	4	1,253 (55.3%)	68 (30.0%)	1,185 (58.1%)	
	5	310 (13.7%)	134 (59.0%)	176 (8.6%)	
	6	12 (0.5%)	10 (4.4%)	2 (0.1%)	

AIS = abbreviated injury scale; GCS = Glasgow coma scale; ISS = injury severity score; rSI = reverse shock index; rSIG = rSI multiplied by GCS score; RTS = revised trauma score; SBP = systolic blood pressure; SI = shock index; TRISS = the trauma and injury severity score.

Table S2. Characteristics variables of patients without traumatic brain injury.

Variables		Total (n = 16482)	Survival No (n = 275)	Survival Yes (n = 16207)	*P*-value
Age (years)		54 [37, 68]	61 [42, 77]	54 [37, 67]	<0.001
ISS		9 [4, 9]	29 [17, 34]	8 [4, 9]	<0.001
GCS		15 [15, 15]	7 [3, 15]	15 [15, 15]	<0.001
SBP (mmHg)		146 [126, 168]	145 [107, 176]	146 [126, 168]	0.019
HR (times/min)		85 [75, 97]	96 [78, 118]	85 [75, 96]	<0.001
RR (times/min)		18 [18, 20]	20 [18, 21]	18 [18, 20]	<0.001
SI		0.58 [0.48, 0.70]	0.67 [0.51, 0.93]	0.58 [0.48, 0.69]	<0.001
rSI		1.73 [1.43, 2.09]	1.49 [1.07, 1.97]	1.74 [1.44, 2.09]	<0.001
rSIG		25.56 [20.82, 31.00]	10.69 [5.09, 20.45]	25.67 [21.00, 31.09]	<0.001
RTS		7.84 [7.84, 7.84]	5.97 [4.09, 7.84]	7.84 [7.84, 7.84]	<0.001
TRISS		0.98 [0.97, 1.00]	0.72 [0.36, 0.94]	0.98 [0.97, 1.00]	<0.001
Sex	Female	7,302 (44.3%)	97 (35.3%)	7,205 (44.5%)	0.003
	Male	9,180 (55.7%)	178 (64.7%)	9,002 (55.5%)	
AIS (Head)	0	13,407 (81.3%)	87 (31.6%)	13,320 (82.2%)	<0.001
	1	1,006 (6.1%)	12 (4.4%)	994 (6.1%)	
	2	388 (2.4%)	6 (2.2%)	382 (2.4%)	
AIS (Face)	0	13,872 (84.2%)	216 (78.6%)	13,656 (84.3%)	<0.001
	1	736 (4.8%)	5 (1.8%)	731 (4.5%)	
	2	1,829 (11.1%)	50 (18.2%)	1,779 (11.0%)	
	3	45 (0.3%)	4 (1.5%)	41 (0.3%)	
AIS (Thorax)	0	14,160 (85.9%)	154 (56.0%)	14,006 (86.4%)	<0.001
	1	337 (2.0%)	5 (1.8%)	332 (2.1%)	
	2	592 (4.0%)	14 (5.1%)	578 (3.6%)	
	3	948 (5.8%)	51 (18.6%)	897 (5.5%)	
	4	419 (2.5%)	43 (15.6%)	376 (2.3%)	
	5	25 (0.2%)	7 (2.6%)	18 (0.1%)	
	6	1 (0.01%)	1 (0.4%)	0 (0.0%)	
AIS (Abdomen)	0	15,223 (92.4%)	210 (76.4%)	15,013 (92.6%)	<0.001
	1	78 (0.5%)	1 (0.4%)	77 (0.5%)	
	2	651 (4.0%)	27 (9.8%)	624 (3.9%)	
	3	366 (2.2%)	14 (5.1%)	352 (2.2%)	
	4	129 (0. 8%)	19 (6.9%)	110 (0.7%)	
	5	35 (0.2%)	4 (1.5%)	31 (0.2%)	
AIS (Extremity)	0	2,901 (17.6%)	96 (34.9%)	2,805 (17.3%)	<0.001
	1	1,034 (6.3%)	2 (0.7%)	1,032 (6.4%)	
	2	7,139 (43.3%)	89 (32.4%)	7,050 (43.5%)	
	3	5,358 (32.5%)	73 (26.6%)	5,285 (32.6%)	
	4	43 (0.3%)	12 (4.4%)	31 (0.2%)	
	5	7 (0.04%)	3 (1.1%)	4 (0.02%)	
AIS (External)	0	14,965 (90.8%)	248 (90.2%)	14,717 (90.8%)	<0.001
	1	1,407 (8.5%)	17 (6.2%)	1,390 (8.9%)	
	2	85 (0.5%)	2 (0.7%)	83 (0.5%)	
	3	12 (0.1%)	0 (0.0%)	12 (0.1%)	
	4	3 (0.02%)	0 (0.0%)	3 (0.02%)	
	5	5 (0.03%)	4 (1.5%)	1 (0.01%)	
	6	5 (0.03%)	4 (1.5%)	1 (0.01%)	

AIS = abbreviated injury scale; GCS = Glasgow coma scale; ISS = injury severity score; rSI = reverse shock index; rSIG = rSI multiplied by GCS score; RTS = revised trauma score; SBP = systolic blood pressure; SI = shock index; TRISS = the trauma and injury severity score.
